# Curcumin, bisdemethoxycurcumin and dimethoxycurcumin complexed with cyclodextrins have structure specific effect on the paracellular integrity of lung epithelia *in vitro*

**DOI:** 10.1016/j.bbrep.2015.11.004

**Published:** 2015-11-10

**Authors:** Berglind Eva Benediktsdottir, Olafur Baldursson, Thorarinn Gudjonsson, Hanne Hjorth Tønnesen, Mar Masson

**Affiliations:** a Faculty of Pharmaceutical Sciences, School of Health Sciences, University of Iceland, Hofsvallagata 53, IS-107 Reykjavik, Iceland; b Department of Pulmonary Medicine, Landspitali-The National University Hospital of Iceland, Eiríksgata 5, IS-101 Reykjavík, Iceland; c Biomedical Center, School of Health Sciences, University of Iceland, Vatnsmýrarvegur 16, IS-101 Reykjavík, Iceland; d School of Pharmacy, Dept. of Pharmaceutics, University of Oslo, Blindern, 0136 Oslo, Norway

**Keywords:** CDs, cyclodextrins, CF, cystic fibrosis, CFTR, cystic fibrosis transmembrane regulator, TER, transepithelial electrical resistance, TJs, tight junctions, Bronchial epithelium, Cyclodextrin, Curcumin, Epithelial integrity, TER, VA10

## Abstract

The phytochemical curcumin may improve translocation of the cystic fibrosis transmembrane regulatory (CFTR) protein in lung epithelium and therefore be helpful in the treatment of cystic fibrosis (CF) symptoms. However, previous studies often use commercial curcumin that is a combination of curcumin, demethoxycurcumin and bisdemethoxycurcumin which could affect the investigated cells differently. In the present study, we investigated the potential difference between curcumin, bisdemethoxycurcumin and dimethoxycurcumin on the epithelial tight junction complex, in the bronchial epithelial cell line VA10, by measuring transepithelial electrical resistance (TER), immunofluorescence and western blotting of tight junction proteins. The curcuminoids were complexed with hydroxypropyl-γ–cyclodextrin for increased solubility and stability. Curcumin (10 µg/ml) increased the TER significantly after 24 h of treatment while four times higher concentration of bisdemethoxycurcumin was required to obtain similar increase in TER as curcumin. Interestingly, dimethoxycurcumin did not increase TER. Curcumin clearly affected the F-actin structures both apically and basolaterally. These results begin to define possible effects of curcuminoids on healthy bronchial epithelia and shows that difference in the phenyl moiety structure of the curcuminoids influences the paracellular epithelial integrity.

## Highlights

• Curcuminoids formulized with cyclodextrin for increased solubility and stability.
• Curcumin increases TER in a concentration dependent manner and causes decrease in apical F-actin staining.
• Higher concentration required for bisdemethoxycurcumin to increase TER compared to curcumin.
• Dimethoxycurcumin did not increase TER.

## Graphical abstract

**Figure:**
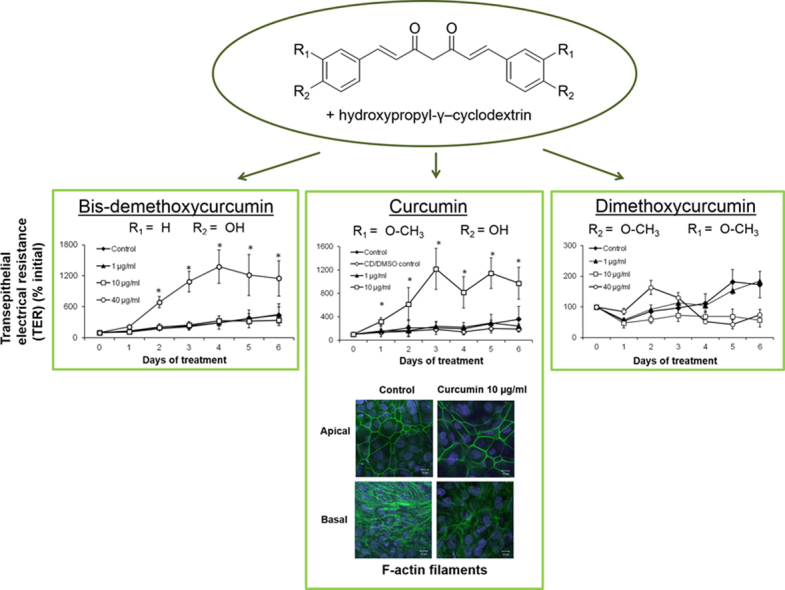

## 1 Introduction

Curcumin is a phytochemical, found in the dried rhizome of the plant Curcuma longa L. The dried rhizome, called turmeric, is often used as spice and is a common ingredient in curry powder. Amount of curcumin in turmeric is commonly around 2–8% [1]. In Southeast Asia, turmeric is not only used as a spice or a coloring agent but is also used to externally treat wounds, inflammation and tumors, liver-and gall diseases among other illnesses. Curcumin has been studied from a pharmaceutical perspective regarding its antioxidant, anti-inflammatory and anti-cancer properties [2]. It is currently one of nearly twenty possible therapies against cystic fibrosis (CF) in development according to Cystic Fibrosis Foundation. A phase I clinical trial, to assess safety and dosage parameters when given to CF patients, has been initiated. CF is a lethal, hereditary disease caused by a mutation in the gene that codes for the cystic fibrosis transmembrane regulator (CFTR) chloride channel protein [3], [4], [5] causing the misfolded CFTR protein to be degraded [6]. This disease is characterized by chronic respiratory infections and inflammation and irrespective of increased knowledge of the CF pathology, the mean predicted survival of CF patients is around 40 years [7]. Studies have shown that if the mutant CFTR protein could relocate from the endoplasmic reticulum to the plasma membrane, it could restore the chloride pump activity [8].

Curcumin may improve the translocation of the CFTR chloride channel protein in lung epithelium [9] although recent studies have not been able to confirm those results [10], [11], [12]. Berger and colleagues discovered that curcumin stimulated the activity of the CFTR channels by elongating the channel opening time and these effects were dose dependent, reversible and ATP dependent [13]. Curcumin also increased the chloride transport of CFTR channels in normal lung epithelia but was unsuccessful in the defected CFTR channels [13]. Similarly, it has been reported that curcumin opens CFTR channels but unlike the study by Berger, the CFTR opening was not dependent on ATP [14].

Tight junctions (TJs) control paracellular ion- and water transport and are necessary for the tightness of the epithelium and are a key part in lung defenses [15]. CF patients often acquire chronic pulmonary infections by the bacteria *Pseudomonas aeruginosa* that disrupts the epithelial barrier integrity [16], [17]. The macrolide antibiotic azithromycin has proven beneficial with CF patients in concentrations not affecting the bacterial count [18], [19]. Interestingly, this macrolide also increased the paracellular integrity in normal bronchial epithelial cells [20] and protected the epithelium during *P. aeruginosa* infection at concentrations not affecting the bacterial count [21]. When considering the potential role of curcumin in CF pathogenesis, it is important not only to consider the CFTR translocation but also the effect on paracellular integrity of normal epithelium.

**Fig. 1:**
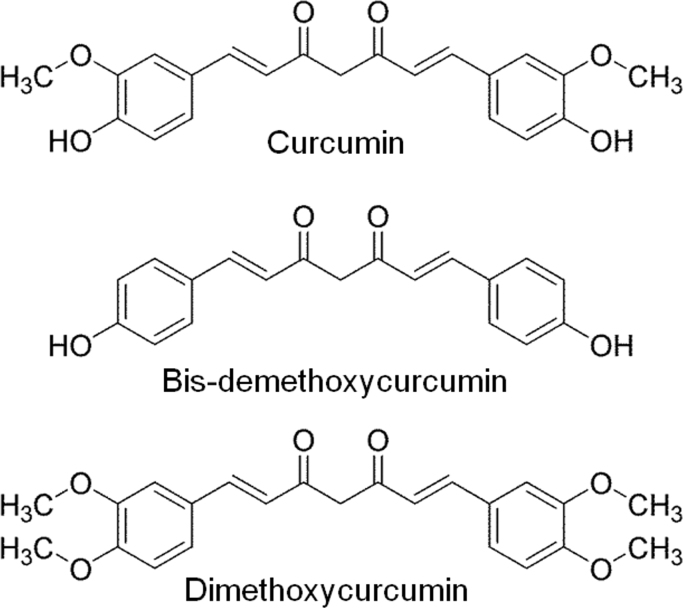
The curcuminoids curcumin, bisdemethoxycurcumin and dimethoxycurcumin have different substitutions on the phenyl ring.

Most studies use commercial grade curcumin [14], [22], [23] which is composed of curcumin (75–85%), demethoxycurcumin (10–20%) and bisdemethoxycurcumin (5%) [24]. As a result, valid concerns arise regarding the use of this curcumin combination since the effects of one curcuminoid could be masked by other curcumin components. Additionally, previous limitations with the use of curcumin is its poor solubility at acid and physiological pH and rapid hydrolysis at basic pH [25]. This results in the use of DMSO and/or ethanol as a solvents [22], [23], [26], which can have adverse effects on the investigated cells [27], [28]. By complexing curcumins with cyclodextrins (CDs) in aqueous solutions [29], [30], the solubility and stability can be increased [30], [31]. Here, we investigate whether curcumin and the curcuminoids dimethoxycurcumin and bisdemethoxycurcumin (Fig. 1), complexed with CDs, increase the paracellular epithelial integrity and if those effects are different between derivatives.

## 2 Materials and methods

### 2.1 Curcuminoid solutions

Curcumin (diferuloylmethane) and curcuminoids (bisdemethoxycurcumin and dimethoxycurcumin) were synthesized and characterized as previously described [31], [32]. The hydroxypropyl-γ–CD (HPγCD) improves the solubility of curcumin and the curcuminoids dimethoxycurcumin and bisdemethoxycurcumin [31] and was therefore chosen as a complexing agent. The stock solutions of the curcumin and the curcuminoids contained 200 µg/ml curcumin compound, 5% v/v dimethylsulfoxide (DMSO) and 15% w/v hydroxypropyl-γ-cyclodextrin (HP-γ–CD, Cavasol® WB HP Pharma, Wacker- Fine Chemicals, Germany) in phosphate buffer saline (PBS) and were all stored away from light in at 5–8 °C. Test solutions were prepared by dissolving appropriate amounts of stock solutions into the DMEM-F12+Ultroser-G cell culture medium. Amount of CD/DMSO solution that curcumin and curcuminoids were dissolved in was also tested for possible effects on TER and TJs.

### 2.2 Cell culture

The newly established and validated bronchial epithelial cell line, VA10 [33], [34] was used between passages 13–20. The cells were maintained in 75 cm^2^ flasks in a humidified incubator at 37 °C (5% CO_2_) containing bronchial epithelial growth medium (BEGM, Cambrex, East Rutherford, NJ, USA). Medium was aspirated and changed every other day with a fresh, prewarmed medium. The cells were seeded at the density of 1×10^5^ cells/cm^2^ on the upper chamber of Transwell filters (pore size 0.4 µm, 12 mm diameter, polyester membrane, Corning Costar Corporation) and cultured in BEGM medium for 5-6 days, with 0.5 ml medium added to the apical side and 1.5 ml medium to the basolateral side. Subsequently, the cells were cultured in Dulbecco’s minimum essential medium Ham’s F12 1:1 (DMEM/F-12) medium (Gibco, Burlington, Canada) supplemented with 2% Ultroser G serum substitute (Pall Life Sciences, Cergy-Saint-Christophe, France) and penicillin/streptomycin. Medium was changed every other day. For morphological analysis and western blot proteins analysis, the cells were seeded on a 6 well plates (Falcon Multiwell 6 Well, Becton Dickinson, NJ, USA) at 2×10^5^ cells/well and cultured in BEGM.

### 2.3 Transepithelial Electrical Resistance (TER) Measurement

TER of VA10 cell layers was measured with Millicell-ERS volthometer (Millipore, MA, USA). The corrected TER value was obtained after subtraction of the background from the cell-free culture insert.

### 2.4 Immunocytochemistry

VA10 cells were fixed for 10 min with 3.7% formaldehyde, permeabilized with 0.1% Triton X-100 for 5 min and then blocked with 10% fetal bovine serum for 5 min. The following primary antibodies were used (diluted in PBS): Mouse anti-human claudin-1 (IgG_1_, 1:125), mouse anti-human ZO-1 (IgG_1_,1:50) and rabbit anti-human occludin (1:20) and were all purchased from Zymed (CA, USA). Cells were incubated with primary antibodies for 30 min followed by incubation with isotype specific Alexa Fluor secondary antibodies (Invitrogen, Oregon, USA,1:1000) and To-Pro-3 (Invitrogen) for nuclear staining (1:500) for 30 min. Alexa Fluor 488 phallotoxin (Invitrogen) was used for F-actin staining (1:40), incubated for 30 min.

### 2.5 Confocal microscope

Immunofluorescence images were obtained using Zeiss LSM 5 Pa confocal laser scanning microscope (CLSM, Carl Zeiss AG, Munich, Germany) with Plan-Neofluar 20×, 40× and Plan-Apochromat 63× oil immersion lenses. VA10 cell layers were mounted with Fluoromount-G (SouthernBiotech, Birmingham, USA) and coverslips before visualization.

### 2.6 Quantification

For quantification of F-actin fluorescence, images were captured with confocal microscopy at the focal plane where F-actin apical staining was most prominent. All images used for quantification were acquired using the same confocal settings. Quantification using immunofluorescence images was performed using Fiji (ImageJ) software.

### 2.7 Western blotting

After the cells grown in 6 well culture plates were treated with PBS, curcumin or curcuminoids, they were lysed in RIPA buffer containing a protease inhibitor cocktail (Aprotinin, PMSF and Na_3_VO_4_). The cells were then scraped from the filters and sonicated for 2 min followed by centrifugation at 12,000×*g* for 20 min at 4 °C. The supernatant was collected and the protein concentration determined with the Bradford assay. Equal amounts of proteins, as determined by the Bradford method [35], were loaded and run on a NuPAGE 10% Bis-Tris gel (Invitrogen, Carlsbad, USA) and transferred to a polyvinylidine difluoride membrane (Invitrogen). The membranes were blocked for 1 h with 5% skimmed milk in 0.25% Tween/PBS and incubated with the primary antibodies mouse anti-E-cadherin (BD Bioscience, IgG2a 1:330), mouse anti-claudin-1 (IgG_1_,1:500) or rabbit anti-occludin (1:1000) overnight, followed by incubation with secondary antibodies (1:2000) for 1 h. Protein bands were visualized using enhanced chemiluminescence system and Hyperfilm (Amersham Bioscience, England).

### 2.8 Morphological analysis

After reaching confluency the cells were incubated with curcumin or curcuminoids. Images were captured on day 1, 4 and 6 after beginning of treatment using Leica DFC320 digital imaging system.

### 2.9 Calculations and data analysis

All data were reported as mean±standard deviation with n representing number of experiments. Unpaired, two tailed Student's *t*-test was done in GraphPad to compare two means with the difference considered to be statistically significant when *p*<0.05.

## 3 Results and discussion

### 3.1 Curcumin and bisdemethoxycurcumin increase transepithelial electrical resistance (TER) significantly in human airway epithelia *in vitro*

**Fig. 2:**
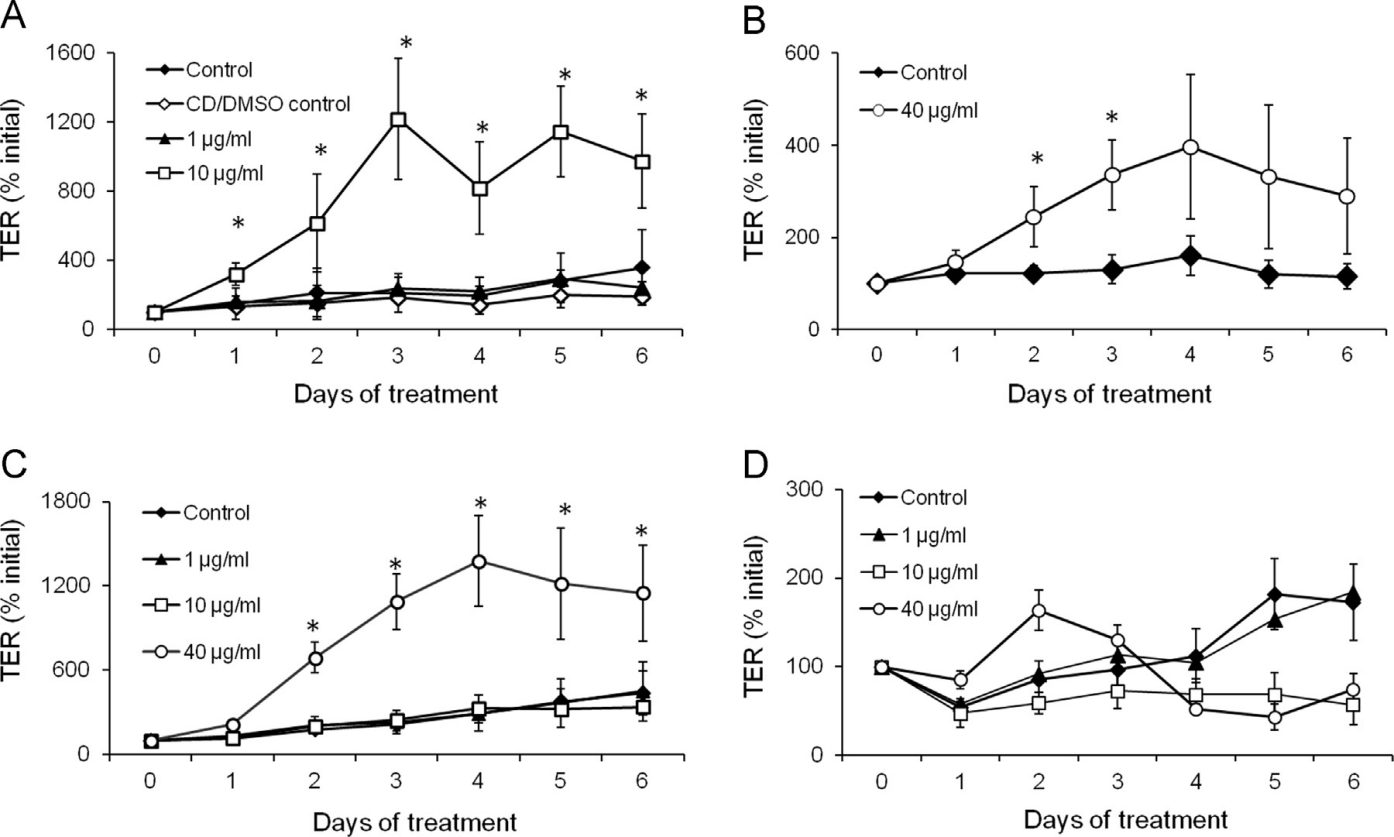
Different effects of curcumin and curcuminoids on TER in human airway epithelia *in vitro*. Human airway epithelial cells were cultured on Transwell permeable support filters. After reaching confluency, curcumin (A, B), bisdemethoxycurcumin (C) or dimethoxycurcumin (D) were added to the basolateral side of the epithelia. TER was measured using a Millicell-ERS electrical resistance system. Data are given as mean±SD (*n*=3).

To determine if the curcuminoids increase the paracellular integrity of epithelia and consequently be beneficial for haltering the CF progression, TER was measured. Indeed, addition of 10 µg/mL curcumin to the basolateral side of the epithelium every other day resulted in significant increase in TER after 24 h treatment (Fig. 2A), 3-fold increase compared to initial value. The increase in TER was not observed in the initial period of the measurements of 60 min (data not shown). After 6 days of treatment, the TER increase had leveled off at around 10-fold increase from initial value. Curcumin at 1 µg/mL did not increase TER as can be seen in Fig. 2A. The increase in TER was not as apparent with 40 µg/mL curcumin, reaching significant higher TER levels compared to control after 5 days of treatment (Fig. 2B). The CD/DMSO control solution did not affect the TER values compared to normal control epithelium (Fig. 2A).

To explore whether the effects of curcumin on TER were specific to the curcumin structure, two other curcuminoids, bisdemethoxycurcumin and dimethoxycurcumin, were investigated (Fig. 2C and D). Interestingly, these curcuminoids had markedly different effects on the bronchial epithelia. Two days of treatment with 40 µg/mL bisdemethoxycurcumin resulted in significant increase in TER that leveled off after 4 days of treatment (Fig. 2C). Conversely, dimethoxycurcumin did not affect the TER values in any of the concentrations investigated (Fig. 2D). After 6 days of treatment with this curcuminoid, the epithelial lining appeared to be less continuous than the control epithelium (Fig. A1), suggesting possible adverse effects on the epithelium. The dose dependent effect of curcumin and the different effects of curcumin and the other curcuminoids on TER measurements indicate a possible agonistic/antagonistic activity that warrants further exploration.

### 3.2 Curcumin affects F-actin localization but not expression of the TJ proteins claudin-1 and occludin and the adherens protein E-cadherin

**Fig. 3:**
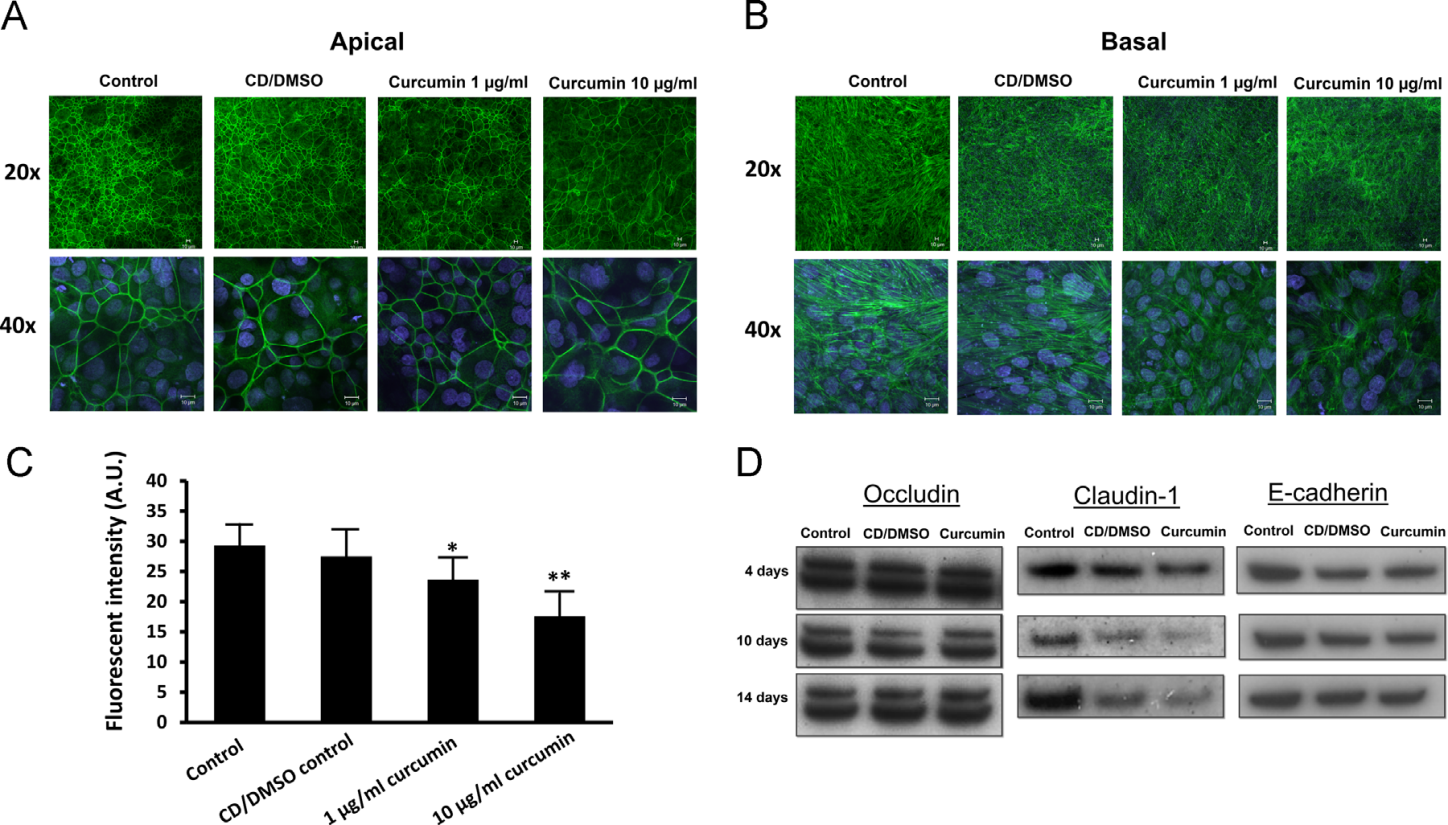
The effects of curcumin on adherens and tight junction proteins.

The TJs are membrane bound proteins that produce apical to basolateral polarity [36] and form a paracellular permeability barrier that limits the permeation to small, uncharged solutes [37]. The TJ complex is dynamic in nature, with its junctional proteins affected by various internal and external stimuli [38]. TER is considered a good indicator of the functional activity of the tight junctions [39]. Since curcumin increased TER, it could possibly affect the expression or localization of TJ proteins or related components. Intracellularly, there are TJ associated proteins such as the members of the ZO family that connect to the actin cytoskeleton which is often affected when the TJ complex is altered (reviewed in [40]). In particular, the actin cytoskeleton is involved in modifications of the tight junction barrier [38] with redistribution of actin filaments been observed to be crucial to the induced barrier formation of endothelial cells by sphingosine 1-phosphate [41]. As can be seen in Fig. 3A and B, altered staining patterns of both apical and basolateral F-actin was observed after treatment with curcumin. The total amount of apical F-actin fibers, as determined by quantification of fluorescence, was significantly reduced after treatment with 1 μg/ml curcumin and this reduction was highly significant after treatment with 10 μg/ml curcumin as Fig. 3C shows. Additionally, basolateral actin was not as filamentous compared to the control epithelium (Fig. 3B). Curcumin is a known upstream inhibitor of NF-κ B [42], a transcription factor that has been shown to interact with the actin cytoskeleton [43], [44]. A possible relationship between the actin rearrangement and increased TER observed in the current study with the known inhibition of the NF-κ B pathway could therefore be possible.

Curcumin affects the localization of F-actin filaments. Bronchial epithelial cells were treated with control, CD/DMSO vehicle or curcumin (1 and 10 µg/mL) for 14 days on Transwell filters. Curcumin at 10 µg/mL clearly affects both apical (**A**) and basolateral (**B**) actin filaments. **C**) Quantification of the F-actin fluorescent intensity at the apical sites shows significant decrease in apical F-actin staining after treatment of 1 μg/ml and 10 μg/ml curcumin. Values represent relative fluorescent intensity in arbitrary units (AU), *n*=5–6 and are expressed as mean±SD. * *p*<0.05 compared to control, ** *p*<0.005. **D**) Expression of occludin, claudin-1 and E-cadherin after curcumin treatment. Bronchial epithelial cells were treated with control, CD/DMSO vehicle or curcumin (10 µg/mL), for 4, 10 and 14 days and the expression determined by western blotting. Claudin-1 expression appears to be gradually changed for both the CD/DMSO control and curcumin treatment.

To further explore these possible effects on the TJ complex, the expression of occludin, claudin-1 and E-cadherin was investigated after treatment with 10 µg/ml curcumin (Fig. 3B). Western blotting of occludin revealed a double band at ~60 kDa but no alterations in the expression at the different time points investigated. E-cadherin is a membrane spanning adhesion protein, essential for formation and maintenance of normal epithelium [45]. Although investigations have indicated that curcumin may down-regulate its expression [46] this was not observed in the current study. Claudins are one of the main components of the tight junction complex that decide both TER and charge specificity [47], [48]. Over twenty claudins have been identified and their expression pattern is specific for each epithelial type [49], [50]. Thus it is indicated that different composition of claudins attribute to different properties of epithelial tissues [47]. Watari and colleagues reported that curcumin (3.7 µg/ml) was a claudin-4 inducer, with concomitant increase in TER of ~170% of initial value after 48 h of treatment [51]. In the current study, both the CD/DMSO vehicle and curcumin (10 µg/ml) appeared to reduce the expression of claudin-1, therefore the direct effects of curcumin on the claudin-1 expression remain inconclusive.

All the curcuminoids had different effect on TER in the bronchial epithelia. Curcumin was more efficient in increasing TER compared to bisdemethoxycurcumin, while dimethoxycurcumin did not affect TER. Rearrangement of the basolateral F-actin stress fibers and its decreased staining at the apical surface was observed after curcumin treatment. These results show that different curcuminoids can have different effects on the epithelia, indicating that future studies would benefit from using pure curcuminoids. The effects of curcumin towards the increased bronchial paracellular integrity could be a part of its beneficial effects in treatment of CF.
