# Supplementary figures and images for: Curcumin, bisdemethoxycurcumin and dimethoxycurcumin complexed with cyclodextrins have structure specific effect on the paracellular integrity of lung epithelia in vitro
Source: Biochem Biophys Rep. 2015 Nov 10;4:405–10. doi: 10.1016/j.bbrep.2015.11.004 (PMC5669517; doi:10.1016/j.bbrep.2015.11.004)

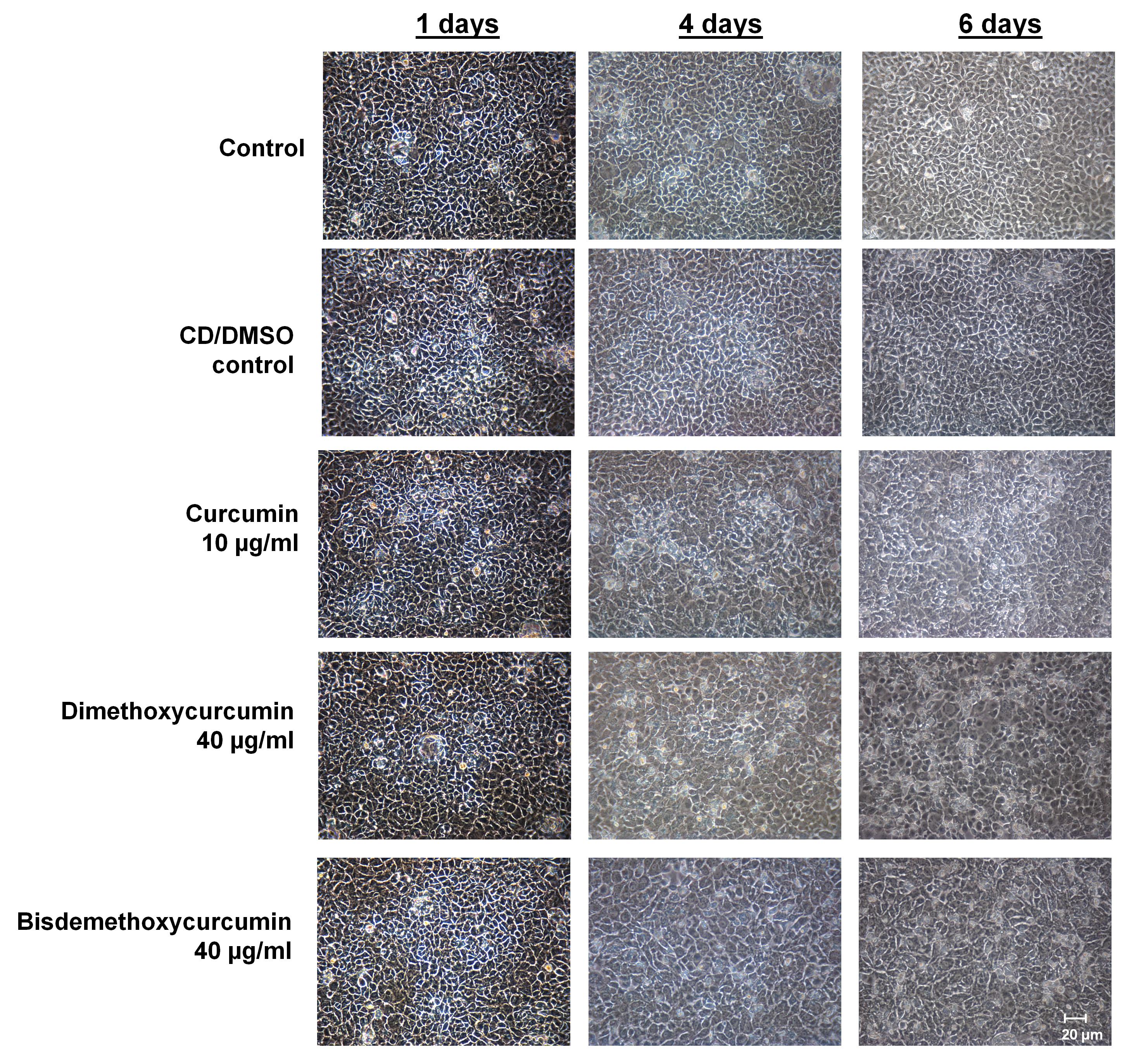

Supplement: Supplementary file 1 — Supplementary material [file mmc1.zip › Supplimentary figure 1.tif]
